# Adenomyosis and its impact on women fertility

**Published:** 2015-06

**Authors:** Elisabetta Garavaglia, Serafini Audrey, Inversetti Annalisa, Ferrari Stefano, Tandoi Iacopo, Corti Laura, Candiani Massimo

**Affiliations:** *Department of Obstetrics and Gynaecology, IRCCS San Raffaele, Via Olgettina 60, Milan, Italy.*

**Keywords:** *Adenomyosis*, *Hyperestrogenism*, *Infertility*, *Pathogenesis*, *Treatment*

## Abstract

Adenomyosis is a widespread disease affecting the reproductive period of women’s life. In the last ten years, different pathogenetic hypotheses have been proposed to explain the initiation and development of the disease. This article aims to present and discuss the most important pathophysiologic mechanisms underlying adenomyosis development in order to clarify the relationship between adenomyosis and infertility. A PubMed search was undertaken for English language literature using the MeSH terms ‘adenomyosis’, ‘infertility’, ‘treatment’, and ‘pathogenesis’. Although the exact etiology of adenomyosis is unknown, many theories have been proposed. We analysed the most important pathogenic theories expressed and evaluated the potential consequences on women fertility. A better comprehension of the adenomyosis pathogenesis has allowed realizing that adenomyosis may affect young women and may have a great impact on their fertility through different mechanisms. The understanding of these mechanisms helps to clarify the potential usefulness of current therapies.

## Introduction

Adenomyosis is a widespread disease, that affects the reproductive period of life. Its prevalence is estimated in literature from 5-70%, and it is difficult to be precisely determined for many reasons. In one third of the cases, adenomyosis is an asymptomatic disease and consequently can be occasionally diagnosed by pelvic ultrasound or it is found in hysterectomy specimens performed for other medical reasons (1). In these cases, diagnosis can be also mistaken from 10-90% among pathologists if histological criteria are not strictly followed. Adenomyosis does not present pathognomonic clinical features: enlarge uterus, dysmenorrhoea (30%), and menorrhagia (50%) can be associated with other diseases (2).

The affection often coexists with other benign disorders, such as endometriosis (70%), leiomyomas (50%), endometrial hyperplasia (35%), endometrial polyps (2%) and endometrial carcinomas (2). In the past, adenomyosis has been attributed to the late reproductive life such as in patients often after their forties and in multiparous women because it was mostly detected in hysterectomy specimens (3, 4). As a matter of fact, from an epidemiological point of view, it has been studied in the “wrong” population. The more and more frequent diagnosis in fertility clinics during the diagnostic work-up and its detection in baboons with lifelong infertility have suggested that it may develop in young women and may have important consequences on fertility (5). In fact, the pathological events determining adenomyosis can appear in the first period of reproductive life, even in adolescence.

In the last ten years, different pathogenetic hypotheses have been proposed to explain the initiation and development of the disease. Their understanding is necessary not only to explain adenomyosis origin but also to clarify its impact on women fertility. Although the proposed mechanisms underlying the disease pathogenesis are based on different pathways, some common concepts can be deduced: adenomyosis is an oestrogen-dependent disease and the endometrial-myometrium interface (EMI) or uterine junction zone (JZ) as a new anatomical and functional entity in uterus wall is mostly likely involved in its development (6).

This article aims to present and discuss the most important pathophysiologic mechanisms underlying adenomyosis development in order to clarify the relationship between adenomyosis and infertility. Novel ideas and aspects might derive from this detailed analysis. A PubMed search was undertaken for English language literature using the MeSH terms ‘adenomyosis’, 'infertility', 'treatment', and 'pathogenesis'.

**Histogenetic hypothesis of adenomyosis origin**

Although the exact etiology of adenomyosis is unknown, many theories have been proposed.

1) Adenomyosis may develop de novo from a metaplastic transformation of the embryologic pluripotent mullerian remnants. Extra uterine sites of adenomyosis such as those observed in the rectovaginal septum support the concept that some cases of adenomyosis may result from this etiologic cause. In addition, studies on the proliferative and biological properties of ectopic and eutopic endometrium has shown that adenomyosis did not respond to the same hormonal changes found in its eutopic endometrium (7).

2) The second hypothesis, expressed by Bergeron *et al* defines ademyosis as an invagination of the basal endometrium into myometrium through an alternated or absent JZ (8). The endomyometrial interface is the only mucous-muscular tissue lacking a distinct, intervening tissue layer, and as a result the endometrium is apposed in direct contact with the myometrium. In this case, the endometrium can slip through bundles of weak smooth muscle fibres that have loosed their tissue cohesion.

The process of invagination and intramyometrial spreading may be facilitated by the non-cyclic, anti-apoptotic activity of the basalis associated with relative hyper-estrogenic states. Estradiol receptor (ER) expression in the adenomyosis foci is greater than in normal endometrium and it is associated with the expression of the apoptosis-suppressing gene product, Bcl-2, throughout the menstrual cycle. The co-expression of Bcl-2 and ER and the hyper-oestrogenic metabolic states may promote both the invagination process and overall ‘spreading’ of adenomyosis into the myometrium. They may also promote other benign disorders: endometriosis, polyps, endometrial hyperplasia and uterine leiomyomas and this is probably the reason for which these conditions are frequently associated with adenomyosis (8).

3) Another hypothesis, expressed by Leyendecker *et al* considers hyper-estrogenic as the leading factor and adenomyosis as a consequence of the mechanism of tissue injury and repair (TIAR) (9). The uterus is constantly active throughout the reproductive period of life and thereby inevitably subjected to mechanical strain. The molecular mechanisms associated with mechanical strain, injury, and repair display a pattern that is quite similar in different tissues and involves the expression of the P450 aromatase and the local production of estrogen (10).

More specifically, it has been suggested that the uterine dysfunction in women with adenomyosis might be the result of local hyperestrogenism while the peripheral estradiol (E_2_) levels are within the normal range (11-15). Local hyperestrogenism leads to increased uterine peristaltic activity of the subendometrial myometrium, imposing supraphysiological mechanical strain on the cells near the fundo-cornual raphe. This state activates the TIAR system focally with further local production of E_2_. The mechanism of tissue traumatization and healing is associated with a specific physiological process that involves the local production of Interleukin-1-(IL-1). IL-1-induced activation of the cyclooxygenase-2 enzyme (COX-2) results in the production of prostaglandin E2 (PGE_2_), which in turn activates the steroidogenic acute regulatory protein (STAR) and the P450 aromatase. Thus, testosterone can be formed and aromatized into E_2_ that exerts its proliferative and healing effects via the ER2 (16, 17).

With a continuing hyperperistaltic activity and sustained injury, healing will not ensue and an increasing number of foci tend to be involved in this process of chronic injury, proliferation, and inflammation. The expansion or accumulation of such sites renders local areas of the basal endometrium to function as an endocrine gland that produces E_2_. Focal estrogen production might reach a tissue level that, in a paracrine fashion, increases uterine peristaltic activity presumably mediated by endometrial oxytocin and its receptor, creating a vicious circle which auto-perpetuates it.

**Figure 1:**
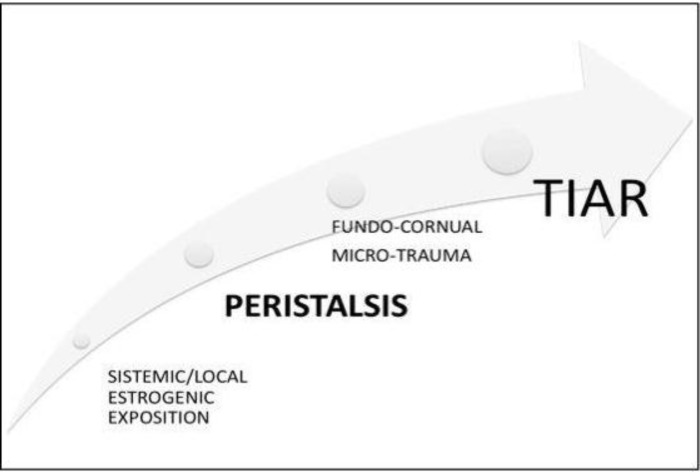
First step: micro-traumatization. The uterus is constantly active thereby subjected to mechanical strain. Molecular mechanisms associated with mechanical strain, injury, and repair displays a pattern which involves the expression of the P450 aromatase and the local production of estrogen. It was suggested that this uterine dysfunction in women with endometriosis and adenomyosis is a result of archimetral hyperestrogenism which leads to increased uterine peristaltic activity of the subendometrial myometrium. Deviations from the normal cyclic endocrine pattern with increases or prolongations of E_2_ stimulation of uterine peristalsis could impose supraphysiological mechanical strain on the cells near the fundo-cornual raphe activating the TIAR system focally with increased local production of E_2_

**Figure 2:**
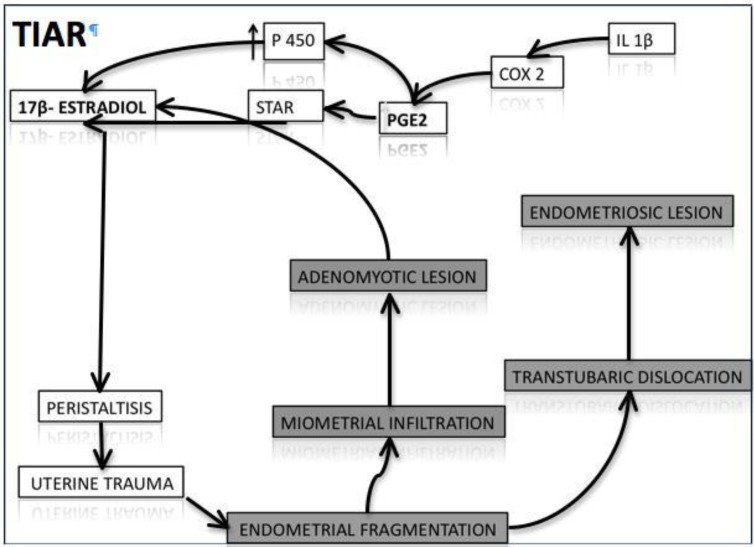
TIAR system: The mechanism of tissue traumatization and healing is associated with a specific physiological process that involves the local production of Interleukin-1-(IL-1). IL-1-induced activation of the cyclooxygenase-2 enzyme (COX-2) results in the production of prostaglandin E_2_ (PGE2), which in turn activates STAR (steroidogenic acute regulatory protein) and the P450 aromatase. Thus, testosterone can be formed and aromatized into E_2_ that exerts its proliferative and healing effects via the ER2. Hyperperistalsis would constitute a mechanical trauma resulting in an increased desquamation of fragments of basal endometrium and, in combination with an increased retrograde uterine transport capacity, in enhanced transtubal dissemination of these fragments. Hyperperistalsis and increased intrauterine pressure would with time, result in myometrial dehiscences that are infiltrated by basal endometrium with the secondary development of peristromal muscular tissue

Hyperperistalsis induced by the local production of estrogen would constitute a mechanical trauma resulting in an increased desquamation of fragments of basal endometrium and in combination with an increased retrograde uterine transport capacity in an enhanced transtubal dissemination of these fragments. Hyperperistalsis and increased intrauterine pressure would with time, result in myometrial dehiscences that are infiltrated by basal endometrium with the secondary development of peristromal muscular tissue. Diffuse or focal adenomyosis of various extents might ensue (11, 13, 15, 18-22) (Figure 1, 2).

4) Another factor that might favour adenomyosis development is represented by the iatrogenic trauma. Surgical interventions might result in extended lesions with an enhanced TIAR reaction (23). The rapidly increasing local estrogen levels during the process of healing interfere with the ovarian control over uterine peristaltic activity leading rapidly to a second step injury with ensuing auto-traumatization and perpetuation of the disease process.

**Alterations in adenomyosis**

**Hormonal alterations**

Most studies performed on adenomyosis share the principle that this disease is primarily characterized by a local hyperoestrogenism, induced by an increase of PGE_2_ (5-9, 11-16, 20-22). This hormonal status represents in turn the “primum movens” of a chain of events that lead to the following alterations. Moreover the hyper-expression of ER induces a down-regulation of progesterone receptors, a loss of their action and finally a progesterone resistance.

**Immunological alterations**

Once the adenomyotic invasion has occurred a crucial question is how adenomyotic foci may elude the immune system. Hyperestrogenism stimulates also the production of IL10 that is an important immunomodulatory cytokine produced by many cell populations (24). Numerous research studies suggest that IL-10 is one of the major anti-inflammatory cytokines and plays important roles in several chronic inflammatory diseases and cancers. Furthermore, uterine IL-10 has been demonstrated to have a dichotomous effect on human leukocyte antigen expression on trophoblast cells by inducing human leukocyte antigen G expression while down-regulating the expression of classical class I and class II antigens (25).

Therefore, Wang *et al* in 2010 postulated that IL-10 expression may contribute to the establishment and maintenance of immunosuppression and might explain the persistence of the ectopic foci within the peritoneal cavity or myometrium without elimination by the immune system of the host. It has been reported that in the eutopic and ectopic endometrium of women with adenomyosis, epithelial cells showed higher expression of IL-10 than in normal controls (24). Moreover, a series of immune responses is activated in adenomyosis, including changes in both cellular and humoral immunity, i.e. a strong expression of cell surface antigens or adhesion molecules, an increased number of macrophages or immune cells, deposition of immunoglobulins and complement components (26-31).

Furthermore, this disease exhibited high frequency of autoantibodies in peripheral blood and in endometrium (26-31). Endometrial cells seem to be under immunological stress, protecting them by exposing heat shock proteins (26-31). An abnormal inflammatory response is also recognized in adenomyosis due to an altered secretion of interleukins -6 and -8 (32). The over expression of IL-6 contributes to the local hyper oestrogenism that is associated with a defect in progesterone receptors and with a reduction of β3 integrin with an altered uterine receptivity. IL-6 is also an “implantation marker” together with Leukaemia Inhibitory Factor (LIF) which is as well modified in women with adenomyosis (33).

An immunological alteration of the uterine environment has been widely demonstrated also by Ota *et al* who described an over-expression of the Human Leukocyte Antigens (HLA) of class II in adenomyosis (26-31). Those molecules are first recognized by macrophages and subsequently activate T cells. Activated T cells secrete cytokine, which in turn stimulate B cells to produce immunoglobulin. Cytokines and immunoglobulin, particularly those against phospholipids (autoantibodies) can induce infertility and miscarriage. Finally the deposition of complement components C3 or C4 in adenomyosis is increased (26-31).

**Increasing in free radicals**

Free radicals are intimately involved in the physiology of reproduction. Enzymes that produce and eliminate free radicals (ie xanthine oxidase, superoxide dismutase, glutathione peroxidase, and nitric oxide synthase) are distributed throughout the body and modulate concentrations of free radicals at an optimal level to maintain the homeostasis (26-31). The overall free radical metabolism is abnormal in adenomyosis. The presence of high levels of intrauterine free radicals has a negative influence during sperm transport, implantation and pregnancy. It has been demonstrated that low concentrations of free radicals are necessary to create an appropriate environment for early embryonic development (34).

Oxygen tension in the fallopian tube or uterus must be markedly lower than that in the atmosphere (60 mm Hg, 30 mmHg vs. 150 mmHg) and embryo can physiologically grow under a reduced-oxygen atmosphere. In this context, many scavenger enzymes in uterus are increased in order to eliminate the excess of free radicals and of nitric oxide (NO), but their hyper activation can produce in turn other free radicals saturating the system. Oxygen free radicals can cause the embryonic arrest and cell death (34). Nitric oxide, a highly reactive free radical, is generated when L-arginine is catalysed to citrulline by a nitric oxide synthase (NOS) and it is known to have different physiologic and pathologic roles in human tissues, modulating uterine contractility during pregnancy and relaxing vascular smooth muscles (35).

Ota *et al* conducted a study in order to evaluate the expression of endothelial nitric oxide synthase in the surface and glandular epithelia of endometrium during the menstrual cycle in endometriosis and adenomyosis and demonstrated that its expression was persistently greater than in control endometrium throughout the menstrual cycle (36). NO is also a powerful inducer of the cyclooxygenase 2 (COX-2), in various inflammation models and therefore elevates local PGE2 and PGI2 concentrations in inflamed tissues, while the inhibitors of NO synthesis block PG production during chronic and acute inflammation (37). It has been postulated that oestrogen is the chief hormonal factor regulating NO production in the reproductive tract, by up-regulating NOS and inhibiting superoxide anion production (38-40).

**Hyper vascularization**

Vascular distribution of the endometrium in adenomyosis has been shown to be different from that of the endometrium of fertile patients without adenomyosis. Hysteroscopy revealed that approximately half of the patients with adenomyosis have an abnormal vascularization: vascular distribution is generally irregular, and vessels were thick, dilated, and/or reticular. Moreover, morphometric analysis of the endometrium revealed that in fertile women, the mean surface area, total surface area, and total number of capillaries are all increased significantly in the secretory phase compared to the proliferative phase (41).

In contrast, the above parameters are increased in the adenomyosis group in both the proliferative phase and secretory phase compared to the fertile women. These findings suggest that regulatory factors involved in the vascular proliferation are diversely exaggerated. Estrogens are supposed to be the most noteworthy factor associated with vascularization acting indirectly on vascular proliferation via growth factors (42). In adenomyosis there are however other factors responsible of an altered endometrial vascularization: a greater activity of the vascular endothelial grow factor (VEGF) and of the hypoxia-inducible factor 1alpha (43-44). 

This determines an abnormal development both in proliferative and secretory phase. A close relationship exists between VEGF and estrogens: an estrogen response element is located upstream of the *VEGF* gene with the consequence that its trascription levels are influenced by estrogens. Indeed, addition of estrogens to human granulosa cells during culture induces VEGF mRNA expression (45, 46). Likewise, VEGF mRNA expression increases when estrogens are added to a human endometrial cell culture medium. Finally, VEGF is rapidly expressed in the endometrium when estrogens are given to ovariectomized rats (42).

**Clinical impact of adenomyosis on fertility**

**Figure 3:**
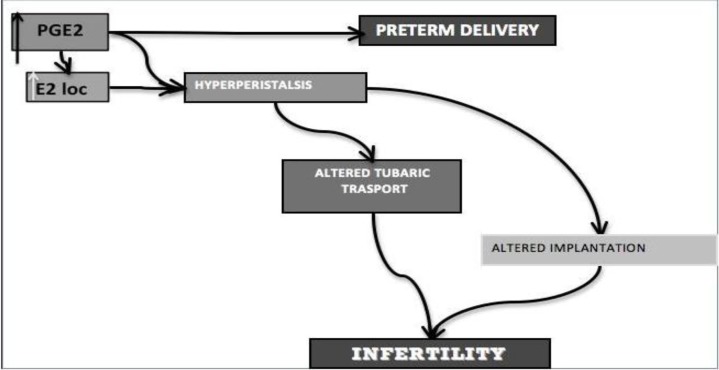
Hyperestrogenism and infertility (A): the TIAR system causes an over-production of PGE2 with an increase in local estrogen concentrations. This may explain the high comorbidity between adenomyosis and endometrial polyps, myomas, endometrial hyperplasia and endometriosis, that in their turn is a cause of female infertility too

**Figure 4:**
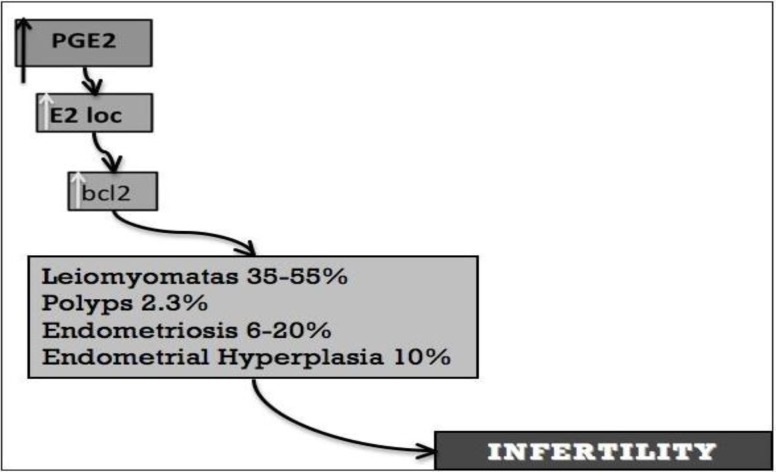
Hyperestrogenism and infertility (B): The increase in PGE-2 and estrogens may lead also to uterine hyper-peristalsis, which has two consequences: an alteration in tubal sperm transportation and an alteration of embryonic implantation. The increase in PGE2 production could also explain the increase risk of preterm delivery in patients with adenomyosis

Adenomyosis may have a negative impact on fertility acting on various mechanisms. The pathogenetic insight underlying this condition is progressively leading to the comprehension of how fertility is impaired by adenomyosis (Figure 3, 4).

**Local hyperestrogenism**

The TIAR system causes an over-production of PGE2 with an increase in local estrogen concentrations. This may explain the high comorbidity between adenomyosis and endometrial polyps, myomas, endometrial hyperplasia and endometriosis, that in their turn is a cause of female infertility. The hyper-expression of ER induces a down-regulation of progesterone receptors and loss of their action with a consequent progesterone resistance. This state contributes to determine an adverse effect on fertilization, implantation and time to pregnancy (11-14).

**Hyper-peristalsis**

The increase in PGE-2 and estrogens may lead also to uterine hyper-peristalsis, which has two consequences: an alteration in tubal sperm transportation and an alteration of embryonic implantation (47). Adenomyosis, especially the diffuse form, is associated with a hyper peristaltic and dysperistaltic uterotubal transport capacity, suggesting that the extent of the adenomyotic component in subjects with endometriosis explains much of the reduced fertility in subjects with an intact tubo-ovarian anatomy.

The high concentration of PGE2 production could also explain the increase risk of preterm delivery and of PPROM in patients with adenomyosis. PG stimulates uterine contractions in vitro and in vivo and control inflammatory responses that result in dilatation and thinning of the cervix (48-50).

**Autoimmune alterations**

The abnormal immune responses associated with adenomyosis might be involved in determining the poor reproductive performance, eventually causing an implantation failure. Activated macrophages producing persistently large amounts of nitric oxide, may impede fertilization and/or implantation. Even after successful implantation, the embryo may be attacked by activated macrophages or T cells, resulting in early miscarriage (27-31).

**Free radicals**

An excessive free radical environment similar to that present in the atmosphere directly may have a negative impact on intracellular DNA and cell membranes, damage fertilized eggs and inhibit embryo development and, thus, pregnancy (27-31). A low-oxygen environment in the uterus must therefore be maintained. In adenomyosis a close cooperation among enzymes, necessary to maintain a defence system against active oxygen, is not achieved. Free radicals can also increase fetal abnormalities.

Jenkinson *et al* reported that the frequency of neural suture deformities increased in a dose-dependent manner when rats were exposed to excessive active oxygen or the injection of nitric oxide donor and nitric oxide synthase inhibitor into the amniotic cavity of rats resulted in malformed embryos (51, 52). Moreover, several studies have indicated that nitric oxide affects human spermatozoa as well. Small amounts of nitric oxide promote human sperm capacitation, whereas a larger amount of nitric oxide seems to reduce sperm motility and induce toxicity. Finally nitric oxide may disturb peristaltic uterine contractions in a paracrine fashion causing uterine hyper peristalsis and dysperistalsis.

**Management strategies**

Therapeutic strategy is directed to interrupt some of those pathological events and it is actually based on anti-oestrogen agents, in order to reduce PGE2 levels and to stabilize immunological environment. In the last 20 years, a variety of drugs have been used to treat adenomyosis and infertility associated with adenomyosis. GnRHa is the first drug used for this purpose, because it decreases expression of aromatase cytochrome p450 and of nitric oxide synthases. As far as the use of progestin concerns, Igarashi *et al* have published interesting results following insertion of an intrauterine system releasing danazol in term of relief from the symptoms and fertility after removal (53).

Also the levonorgest-releasing intrauterine system has been used for the relief of symptoms associated with adenomyosis, but it is not known yet whether the system may be useful in infertile patients (54). Aromatase inhibitors (AI), as anti-oestrogens drugs, may offer some advantages in the treatment of adenomyosis. Kimura *et al* verified that concomitant use of GnRHa and AI was an effective treatment of choice for adenomyosis cases resistant to conventional treatment methods or for women rejecting surgery (55).

Badawy *et al* have confirmed that aromatase inhibitors are as effective as gonadotropin-releasing hormone agonists in reducing adenomyoma volume and improving symptoms (56). Several studies about uterine-artery embolization have underlined a possible approach in the treatment of symptoms associated with adenomyosis (57). Although Pron *et al* has expressed an age-related impairment of ovarian function following uterine artery embolization leading to amenorrhoea, they concluded that pregnancies after uterine-artery embolization are possible, but with a risk of obstetric complications, possibly due to abnormal placentation (59). Finally, the MRI-assisted high-intensity focused ultrasound (HIFU) ablation is a possible new strategy in the treatment of adenomyosis especially in the nodular disease.

## Conclusion

A better comprehension of the adenomyosis aetiology and pathology has allowed realizing that adenomyosis may affect young women and can have a great impact on their fertility through different mechanisms. The understanding of the pathogenetic mechanisms underlying this condition helps to clarify the potential usefulness of current therapies such as local progesterone given as anti-oestrogen agent, and at the same time can suggest new therapeutic approaches. Since the major adenomyosis alterations are intrauterine, a local therapy, released for example by an intra-uterine device, may represent the best choice.

The drug administered may be chosen among the anti-oestrogens, such as aromatase inhibitors or among the anti-inflammatory category, in order to reduce PGE2 levels and to stabilize the immunological environment. In order to reduce the hyper-peristalsis, patients could take advantage from a local muscle-relaxant. Finally, in order to reduce the free radicals patients could receive an anti-oxidant therapy such as vitamin E.

## Conflict of interest

The authors report no conflicts of interest. The authors alone are responsible for the content and writing of the paper.
